# In Vitro Study of Cricket Chitosan’s Potential as a Prebiotic and a Promoter of Probiotic Microorganisms to Control Pathogenic Bacteria in the Human Gut

**DOI:** 10.3390/foods10102310

**Published:** 2021-09-29

**Authors:** Carolyne Kipkoech, John N. Kinyuru, Samuel Imathiu, Victor Benno Meyer-Rochow, Nanna Roos

**Affiliations:** 1 Department of Food Science and Technology, Jomo Kenyatta University of Agriculture and Technology, P.O. Box 62000-00200, Nairobi, Kenya; carolyne.kipkoech@bfr.bund.de (C.K.); jkinyuru@agri.jkuat.ac.ke (J.N.K.); samuel.imathiu@jkuat.ac.ke (S.I.); 2 Federal Institute of Risk assessment, D-12107 Berlin, Germany; 3 Department of Plant Medicals (Agricultural Science and Technology), Andong National University, Andong 36729, Korea; 4 Department of Genetics and Ecology, Oulu University, SF-90140 Oulu, Finland; 5 Department of Nutrition, Exercise and Sports, University of Copenhagen, Rolighedsvej 26, 1958 Frederiksberg, Denmark; nro@nexs.du.dk

**Keywords:** human gut bacteria, growth inhibition, chitin, chitosan, pathogenic, diet, pre- and probiotics

## Abstract

In this study, cricket chitosan was used as a prebiotic. *Lactobacillus fermentum, Lactobacillus acidophilus*, and *Bifidobacterium adolescentis* were identified as probiotic bacteria. Cricket chitin was deacetylated to chitosan and added to either De Man Rogosa and Sharpe or *Salmonella/Shigella* bacterial growth media at the rates of 1%, 5%, 10%, or 20% to obtain chitosan-supplemented media. The growth of the probiotic bacteria was monitored on chitosan-supplemented media after 6, 12, 24, and 48 h upon incubation at 37 °C. Growth of *Salmonella typhi* in the presence of probiotic bacteria in chitosan-supplemented media was evaluated under similar conditions to those of the growth of probiotic bacteria by measuring growth inhibition zones (in mm) around the bacterial colonies. All chitosan concentrations significantly increased the populations of probiotic bacteria and decreased the populations of pathogenic bacteria. During growth, there was a significant pH change in the media with all probiotic bacteria. Inhibition zones from probiotic bacteria growth supernatant against *Salmonella typhi* were most apparent at 16 mm and statistically significant in connection with a 10% chitosan concentration. This study suggests cricket-derived chitosan can function as a prebiotic, with an ability to eliminate pathogenic bacteria in the presence of probiotic bacteria.

## 1. Introduction

Crickets are edible insects and farms to rear them commercially have been established in several countries around the world in recent years [1]. The main objectives for farming crickets have been their nutritive value and their therapeutic roles, for example lowering blood pressure and exerting anti-aging effects [2,3]. Since only a small fraction of humans, according to Paoletti et al. [4] possesses the enzyme chitinase, the chitin cuticle of these insects has generally been regarded as not very useful. Although chitosan, a polymer with applications in food [5], cosmetics [6], biomedical and pharmaceutical realms [7,8], can be obtained from crickets, it is commercially acquired mainly from crustaceans. In this paper, we focus on cricket chitin in connection with pre-and probiotics and show that this component of the cricket body when ingested can exert a beneficial influence on the gut flora of human consumers and need not be regarded as useless [4,9,10].

Prebiotics are fermentable fibres that can benefit the growth of beneficial bacteria in the host’s colon [10]. Positive alteration of the composition and metabolic activity of the host colon is of great interest to human health promoters owing to the important role of the intestinal micro-flora to synthesize vitamins and stimulate the growth of bifidobacteria and lactobacilli. Gut beneficial bacteria, widely referred to as probiotics, have been defined as live microorganisms that positively affect the host’s organism by improving the intestine’s microbial balance [10]. Probiotic bacteria are known to suppress the growth of pathogenic bacteria by lowering the pH and by producing growth-suppressing metabolites. In this way they protect an organism against gastrointestinal illnesses [11].

Recent advances in understanding the important role of prebiotics include demonstrations of prebiotics to stimulate the growth of beneficial bacteria and to alleviate depression by reversing the pathophysiology of depression [12]. Consuming crickets has been shown to promote the growth of probiotic bacteria with reduced plasma TNF-α [13]. In a randomised control trial on the use of oligosaccharide as a prebiotic and *Bifidobacterium lactis* as a probiotic in infants, higher *Bifidobacterium* and *Lactobacillus* with lower clostridia counts occurred in the group that consumed prebiotics [14].

## 2. Materials and Methods

### 2.1. Preparation of Chitosan

Chitosan was obtained by deacetylation of chitin obtained from crickets of the species *Scapsipedus icipe*, farmed at Jomo Kenyatta University of Agriculture and Technology Farm (JIF); latitude 1.10325 S1°6′11.718″, longitude 37.0208 E 37°1′14.898″. A colony was initiated using 625 juveniles and 466 adult crickets (348 females and 118 males). Wild-caught juvenile and adult crickets were transferred separately into transparent Perspex cages (65 cm height × 50 cm width × 65 cm length) with vertically arranged cardboard egg trays to provide hiding sites for the crickets. Each cage had a rectangular opening (20 cm × 35 cm) made on the lid of the cage to which a net was fixed. Two additional openings (25 cm diameter) were also made on the front and backsides of the cage, screened with a net to allow for air circulation.

According to the protocol of Magara et al. [15], the crickets were fed a mixture of soybean flour, wheat bran, and maize diets daily. Besides, fresh plant leaves were also provided regularly. Wet cotton balls with approximately 60% moisture [confirmed using a moisture sensor with two 12-cm-long probes; HydroSenseTM CS620, Campbell Scientific, Inc., Logan, UT, USA] were introduced into the cages to provide water and to serve as oviposition sites for adult crickets. The cotton balls were replaced every 3 days. The colonies were maintained at 27 ± 1 °C, relative humidity (RH) of 65 ± 5%, and a photoperiod of 12:12 (L: D) h cycle.

This species of cricket typically undergoes 9–10 moults to maturity depending on the temperature. In the adult cages, the cotton balls were checked daily, and those containing eggs were carefully removed and transferred into transparent rectangular plastic containers (20 × 15 × 15 cm; Kenpoly Manufacturer Ltd., Nairobi, Kenya). Thereafter, the containers were placed in a climate-controlled chamber at 30 °C with an RH of 70% and a photoperiod of 12:12 (L: D) h light cycle. An opening (15 × 8 cm) was made on the lid of each container and covered with fine netting organza material capable of retaining emerging nymphs. The newly hatched nymphs were transferred into Perspex cages as described above and fed powdered soybean and maize diets ad libitum [15]. The rearing cultures were monitored daily to record and remove dead crickets. The colony was reared for 8–12 generations before the start of the experiment. Once every 6 months, wild-caught crickets were reared separately and the young neonates were transferred to cages, holding the newly hatched neonates of the stock culture to maintain the genetic vigour of the colonies and prevent inbreeding depression as well as disease transfer. In addition, cricket populations were kept at low densities to reduce the stressful crowding effect, which is very common in insect mass production [16].

Although not as well known as *Acheta domesticus* and *Gryllus bimaculatus*, general biology and life cycle of *Scapsipedus icipe* Hugel & Tanga have been described in detail by Otieno et al. [17] and are largely similar to the other commercially used cricket species.

Before the commencement of the experiment, the rearing room was maintained at 27±1°C using Xpelair heater: WH30, 3 KW Wall Fan Heater, and UK. The RH in the experimental room was maintained at 65 ± 5% using a diabatic atomizer humidifier Condair ABS3 and a photoperiod of 12:12 (L: D) h. The condition of the room was monitored daily using a digital thermohydrometer (Humidity/Temperature Traceable Dew Point Meter–4800 CC). From the adult cricket stock colony, eggs (~1 h old) were collected using Petri dishes (9 cm diameter × 1.2 cm height) filled with 70% moist autoclaved wood shavings (sawdust) screened with aluminum wire mesh netting (2 mm^2^) to avoid cannibalism. The eggs were individually counted with the aid of entomological tweezers and a moist fine camel’s hair brush under stereomicroscope (Leica MZ 125 Microscope; Leica Microsystems Switzerland Limited), fitted with Toshiba 3CCD camera using the Auto-Montage software (Syncroscopy, Synoptic Group, Cambridge, UK) at a magnification of 25× to avoid damage.

In total, 3000 eggs were subdivided into three groups (1000 each) and transferred into 4-L transparent rectangular plastic containers (21 × 14 × 15 cm; Kenpoly Manufacturer Ltd., Nairobi, Kenya) containing moist wood shavings (sawdust). The experimental setup was monitored at 6-h intervals daily until eclosion from the eggs commenced. An opening (14.5 × 8.3 cm) was made on the lid of each container and covered with fine netting organza material capable of retaining emerging nymphs. Crickets were sampled three times weekly from the JIF production site, from week 4 to week 13 of the cricket production period. The crickets were cleaned and oven-dried at 50 °C for 72 h and crushed to obtain cricket flour for chitosan extraction.

### 2.2. Extraction of Chitin and Chitosan

The extraction process of chitosan involved three major steps such as demineralisation, deproteinisation, and deacetylation.

#### 2.2.1. Demineralisation

This step was performed using dilute hydrochloric acid (HCl) solution. The raw materials were ground to 20 mesh in a Wiley mill. The samples were demineralised with 2 N HCl at room temperature for 6 h and then were treated with 2 N HCl at 10 °C. In this case, the ratio of the raw material to 2 N HCl was 1 g/10 mL. Then, the samples were washed with distilled water and dried in an oven at 40 °C overnight to eliminate the calcium carbonate and calcium chloride, which constitute the main inorganic compounds of the crickets’ exoskeleton. During the digestion reaction, the emission of carbon dioxide (CO_2_) gas is an important indicator of the content of the mineral materials. The resulting materials were then filtered, washed to neutrality with distilled water, and dried in an oven overnight at 50 °C.

#### 2.2.2. Deproteinisation

The samples were treated with 4% NaOH at 10°C for 24 h and then were washed with distilled water. In this case, the ratio of the sample to 4% NaOH was 1 g/10 mL. Deproteinisation was performed using alkaline treatment using dilute sodium hydroxide (NaOH) solution to remove proteins. The mixture was filtrated, washed several times with deionised water to remove the excess of NaOH, and then dried in an oven overnight. The product obtained was designated as purified chitin.

#### 2.2.3. Deacetylation

This step was to convert chitin to chitosan by removal of the acetyl group. The preparation of chitosan was generally achieved by treatment with concentrated NaOH solution at elevated temperature. After the reaction, the material produced was washed several times with distilled water until neutrality and then dried in an oven overnight.

### 2.3. Structural Characterisation of Chitin

After being dried completely at 50 °C under a vacuum, the sample was used for analysis. FTIR spectra were obtained with a Shimadzu FTIR 8700 spectrophotometer (Tokyo, Japan) under dry air at room temperature with KBr pellets. The pellets were prepared via the thorough mixing of KBr (300 mg) and chitin (3 mg). Solid-state CP–MAS ^13^C NMR spectra were obtained at a ^13^C frequency of 500 MHz with a Bruker Avance-300 NMR spectrometer. Spectra were acquired with a contact time of 0.224 s. A repetition time of 10 s was used for all the samples. The spinning speed was 8567 Hz, and the number of scans was 876.

### 2.4. Antibacterial Activity and Mechanism of Chitosan

#### 2.4.1. Microorganisms and Cultivation

All microbiological media and chemicals were obtained from Sigma Chemical Company Ltd. unless otherwise indicated. Commercial media for probiotic bacteria, de Man, Rogosa, and Sharpe Agar, and broth (MRS agar and broth) were used. For *Salmonella typhi*, *Salmonella/Shigella* (SS) agar was used. For the combined cultivation of probiotics and pathogenic bacteria, a commercial nutrient broth was used. Chitosan-amended media were prepared by substituting SS or MRS bacterial media with 1%, 5%, 10%, or 20% chitosan. The control medium consisted of media without chitosan, as either MRS, nutrient broth, or *Salmonella/Shigella* agar, which before use was sterilized at 121 °C for 15 min. Probiotic bacterial cultures: *Lactobacillus fermentum* ATCC 9338, *Lactobacillus acidophilus* ATCC 4356, and *Bifidobacterium adolescentis* ATCC 15703 were obtained from Chr. Hansen-Denmark through Promaco Ltd., Nairobi, Kenya. The pathogenic bacterium *Salmonella typhi* ATCC 6539 was obtained from Kenya Medical Research Institute, Nairobi, Kenya.

#### 2.4.2. Antibacterial Assessment

Antibacterial activities of the series of chitosan and its derivatives against *Salmonella typhi* were evaluated. A representative colony was picked off with a wire loop and placed in a nutrient broth (peptone 10 g, beef extract 3 g, NaCl 3 g in distilled water 1000 mL; pH 7.0), which was then incubated at 37 °C overnight. Then, a culture where *S. typhi* grew in a logarithmic growth phase was prepared for an antibacterial test. 0.1 g of chitosan was dissolved in 4.9 g of nutrient medium containing 0.1 mol/L of acetic acid. After chitosan was completely dissolved, it was gradiently diluted by 5.0 g of nutrient medium to chitosan concentrations of 1%, 5%, 10%, and 20%. Its pH value was adjusted to 6.0 with dilute NaOH solution. These test tubes containing nutrient medium and chitosan were sterilised at 121 °C for 15 min. After cooling down, 50 µL of bacterial suspension was added to above each test tube and cultured at 37 °C for 24 h. The controlled test tube contained the nutrient medium (pH 6.0) with bacterial suspension but without chitosan. Then 0.1 mL of bacterial suspension was transferred to an agar plate (three plates for one sample) and cultured at 37 °C for 24 h.

A loopful of each culture was spread to obtain single colonies on the nutrient agar (agar 15 g, peptone 10 g, beef extract 3 g, NaCl 3 g in distilled water 1000 mL; pH 7.0) and incubated at 37 °C for 24 h. Colonies were then counted and colony-forming units calculated. Enumeration of bacteria was done after an incubation at 37 °C for 6 h, 12 h, 24 h, and 48 h, where 0.1 mL of 10^−6^ of each replicate was pour-plated and cultivated on MRS agar and *Salmonella/Shigella* (SS) agar for cell colony counts and calculation of cell colony-forming units. The number of colonies was read and the average value was obtained. The control was the bacterial suspension with the same pH value but without chitosan. The bactericidal rate (R) of every sample was calculated according to the following Formula: R = B − A B × 100. A was the number of colonies of the tested plate (CFU/mL); B was the number of colonies of the controlled plate (CFU/mL).

To determine the growth of pathogenic and probiotic bacteria on different concentrations of chitosan, 0.1 mL samples of 16-h old bacterial cultures were incubated in nutrient broth with different concentrations of chitosan 1%, 5%, 10%, or 20%, incubated at 37 °C for 6 h, 12 h, 24 h, and 48 h. The cultures were serially diluted 10-fold and 0.1 mL of 10^−6^ dispensed and pour-plated on either SS agar or MRS agar and incubated at 37 °C for 48 h; the colonies were counted and colony-forming units (CFUs) calculated. Before each incubation, the pH was adjusted to neutral by the use of sodium hydroxide or hydrochloric acid and monitored during the experiment period.

#### 2.4.3. Chitosan Inhibition of *S. typhi* Growth

The plate well diffusion method [18], was used to visualize the formation of a zone of inhibition in a solid culture medium, i.e., *Salmonella/Shigella* (SS) agar plates. The procedure carried out and used in this analysis follows the agar diffusion method, in which small circular cavities are punctured in the culture medium and filled with approximately 0.25 mL of chitosan for each concentration. Then 50 µL of bacterial suspension was spread and the plates were stored for 24 h at 37 °C to allow growth. Inhibition zones were measured in mm based on the average diameter of the clear area, directly on the dishes [19].

### 2.5. Data Analysis

Data collected were recorded in Microsoft excel^®^ 2016 (16.0.5188.1000). Data normality was tested using the Kolmogovov—Smirnov test. Statistical analyses of the data were performed using the statistical methods of Motulsky [20]. Data are presented as means ± standard deviation (SD) and were analysed by one-way ANOVA followed by Tukey’s multiple comparisons test across experimental groups. The difference between means was considered significant at *p* ≤ 0.05. In the result and discussion section, the word ‘significantly’ is used to denote the statistically significant difference. For each species as well as for the sum of pathogenic and probiotic species, a linear fixed model with experimental run as a random factor was applied. In all cases, comparisons with the control were set up and corrected for simultaneous hypothesis testing according to Dunnett.

## 3. Results

**Figure 1:**
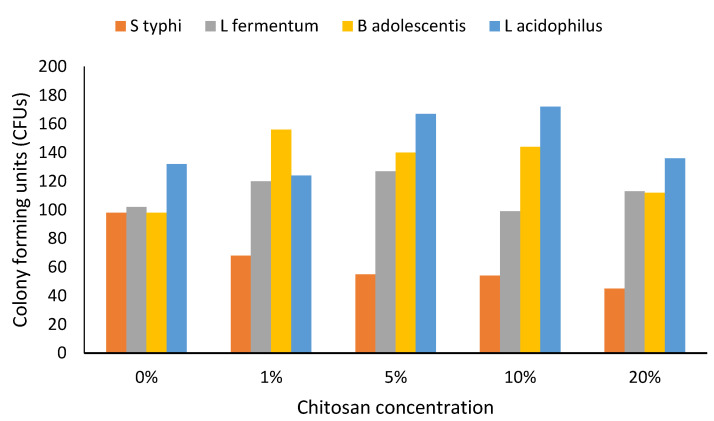
Growth of bacterial cells in chitosan-amended media (24 h). *n* = 3. Abbreviation: S typhi: *Salmonella typhi*, L fermentum: *Lactobacillus fermentum*, B adolescentis: *Bifidobacterium adolescentis*, L. acidophilus: *Lactobacillus acidophilus.* Bacterial growth in 0%, 1%, 5%, 10% and 20% chitosan-supplemented media was monitored at 24 h. Bacterial growth was measured in colony-forming unit per millilitre (CFU/mL).

The growths of *Salmonella typhi* and probiotic bacteria at different chitosan concentrations are shown in Figure 1. The bacterial growth proceeded as per the expected bacterial curve, except that when chitosan concentration in the media was increased, the growth of the pathogenic bacteria slowed down while the growth of the probiotic bacteria (especially that of *L. acidophilus*) expanded (Figure 1).

**Figure 2:**
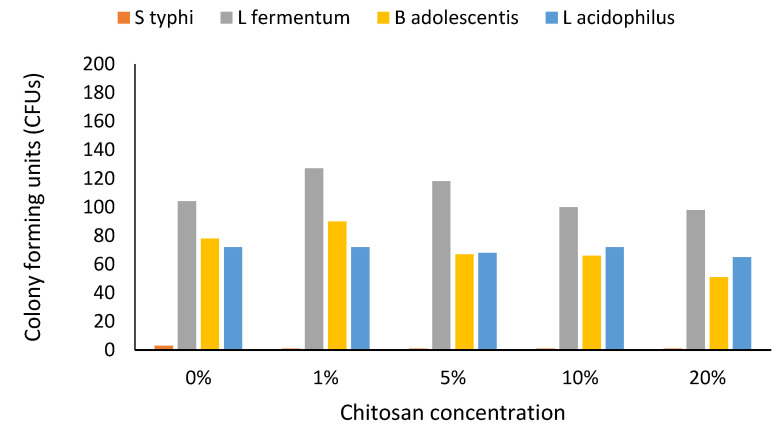
Effect of bacterial growth on chitosan-amended media in the presence of probiotic-prebiotic at a ratio of 1:1 (24HRS). *n* = 3. Abbreviation: S typhi: *Salmonella typhi*, L fermentum: *Lactobacillus fermentum*, B adolescentis: *Bifidobacterium adolescentis*, L. acidophilus: *Lactobacillus acidophilus.* Bacterial growth in 0%, 1%, 5%, 10% and 20% chitosan-supplemented media was monitored at 24 h. Bacterial growth was measured in colony-forming unit per millilitre (CFU/mL).

Combined chitosan and probiotic bacteria effects on the growth of the pathogenic bacteria were assessed. An increase in growth was seen in the first 6 h before a drop in *Salmonella typhi* was noted (Figure 2). Even in media that did not contain chitosan but contained the probiotic bacteria, *Salmonella typhi* growth was highly and severely suppressed at 24 h; suppression time was shortened with increased chitosan concentration (Figure 2).

**Figure 3:**
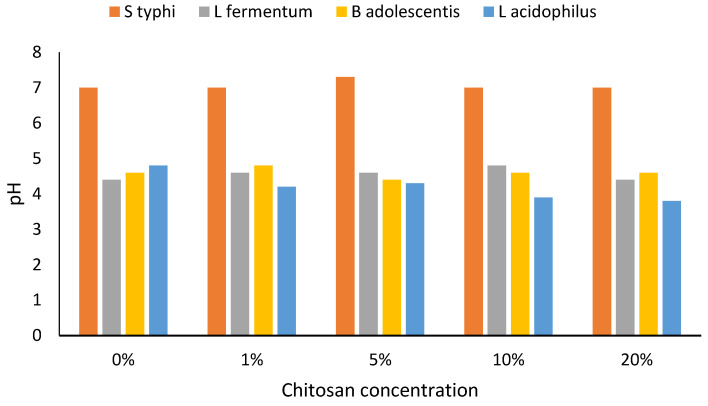
Change of pH in chitosan-amended media during bacterial cell growth (24 h). *n* = 3. Abbreviation: S typhi: *Salmonella typhi*, L fermentum: *Lactobacillus fermentum*, B adolescentis: *Bifidobacterium adolescentis*, L. acidophilus: *Lactobacillus acidophilus*.

As the probiotic and pathogenic bacterial cells grew, the pH fell to around 4, being initially neutral. However, differences in connection with different chitosan concentrations were apparent (Figure 3).

When probiotic bacteria were cultivated on chitosan-supplemented media and the supernatant of the growth media was used to inhibit the growth of pathogenic bacteria, inhibition zones were seen even in the media that did not contain chitosan (although this inhibition did not reach statistical significance: *p* = 0.05; M = 10 mm, SD = 0.3). The highest and significantly most different inhibition was apparent in connection with the 16 mm zone seen in *L. fermentum* at a 10% chitosan concentration (Table 1). An increase in chitosan concentration to 20% also led to an increased diameter of the inhibition zone in *B. adolescentis*.

## 4. Discussion

### 4.1. Chitosan and Chitin Characteristics

Chitosan is a natural antimicrobial agent found in the shells of crustaceans: majorly crabs, shrimp, and crayfish, and some studies have pointed to the possibility of chitosan production from squid and fungi [21]. In this study, chitosan was extracted from edible insects. There was a steady increase in bacterial growth between 0 and 12 h, which remained stable up to 48 h before dropping, following an expected normal bacterial growth curve. The lowest increase in growth was seen at 20% chitosan concentration especially in *Salmonella typhi*, this could be due to the depletion of available nutrients in the growth media and an inability of *Salmonella typhi* to ferment chitosan and utilize it for its growth. At 48 h in 20% chitosan concentration, all bacterial cells were suppressed with bacterial cells reduced to the initial number at 0 h. There was no significant change in *Salmonella typhi* growth in chitosan-supplemented media, and the normal bacterial growth curve was lacking, which may indicate that *Salmonella typhi* growth was suppressed by the presence of chitosan. In the absence of chitosan, *Salmonella typhi* growth was significant at 12 h. *Salmonella typhi* growth was limited by an increased concentration of chitosan, probably because *Salmonella typhi* was unable to break down chitosan. That is what could have affected the availability of favourable growth media and led to a limitation of nutrients amid increased bacterial concentration.

The exact mechanism of chitosan’s antibacterial activity is yet to be fully understood. It is known, however, that chitosan’s antimicrobial activity is influenced by several factors that mainly act in an orderly, yet independent way. Sea creatures have been the main sources of chitin and its derivative chitosan; insects are a new potential source [22]. Chitin yield, molecular weight and the degree of deacetylation determine its properties, [6,7,23,24] and insects have been shown to have a higher deacetylation percentage. The most likely antibacterial activity of chitosan is by binding to the negatively charged bacterial cell wall, disrupting the cell and, thus, altering the membrane permeability, followed by attachment to DNA causing inhibition of DNA replication and subsequently cell death. Another possible mechanism is that chitosan acts as a chelating agent that selectively binds to trace metal elements causing toxin production [21]. It could be postulated that chitosan disrupts the barrier properties of the cell wall structure of Gram-negative bacteria, as advanced by the measurement of a potassium release [25]. This mechanism could be explained by the presence of the free amino groups from the chitosan structure, causing variable mortality rates in different bacterial strains [26].

The probiotic bacteria were likely able to ferment chitosan in the media and then continue to use it for their normal growth. *Lactobacillus fermentum* and *Lactobacillus acidophilus* exhibited the highest growth although the growth of *Lactobacillus acidophilus* initially picked up slowly. This shows that probiotic bacteria can degrade chitosan only to a limited concentration and beyond this; higher chitosan concentration may not be beneficial in connection with the growth of probiotic bacteria. Past studies have shown prebiotics such as inulin and chitin can restrict the growth of pathogenic bacteria [14,27].

The gut microbiome plays an important role in the health of humans and animals. Beneficial microbes diversity can be modulated by diet [28]. Fermentable sources of fibre, and in particular insect chitin, often increase the abundance of beneficial microbes. This study demonstrates that chitosan can increase the growth of probiotic bacteria, but that its usefulness as a prebiotic to profoundly modify the gut microbial composition depends on an optimal concentration for beneficial bacteria to be able to ferment this carbohydrate. When food ingested contains chitin, which can pass through the digestive system and get to the colon almost unaltered, beneficial bacteria that reside in the colon can ferment and deacetylate [29] and utilise the products for their growth [30]. Chitin may be introduced through consumption of whole edible insect products, or in food as an additive to normally consumed food such as yogurt that may already contain probiotic bacteria.

### 4.2. Interactions

When *Salmonella typhi* and probiotic bacteria were cultivated in chitosan-supplemented media in the ratio of 1:1, the drop in bacterial cell growth paralleled chitosan concentration increases; only *Lactobacillus fermentum* seemed to thrive in high chitosan concentrations and the presence of the pathogen in the media (Figure 3). The numbers of colony-forming units became reduced in *Salmonella typhi* after 6 h of growth. *Bifidobacterium adolescentis* and *Lactobacillus acidophilus* growth in different concentrations did not change much over time. This was a different scenario from the initial growth of probiotic bacteria in chitosan-supplemented media where there was a significant increase in growth. This could have been due to the presence of *Salmonella typhi,* which was probably exerting a negative impact on probiotic bacterial growth as a survival strategy. Pathogenic bacteria have over time developed means of survival in the gut environment [31].

Probiotic bacteria grew well in chitosan-supplemented media, although the growth was slowed by the presence of *Salmonella typhi*, possibly due to a lack of sufficient media and unknown effects of *Salmonella typhi* exerted as a survival strategy, which may likely include toxins as a survival strategy [32]. On the other hand, in *Salmonella typhi*, populations were greatly reduced, which could have been caused by the combined effects of an inability to ferment chitosan and unknown factors involving products of the probiotics as part of a survival strategy. Recent studies have shown probiotic bacteria to be able to suppress pathogenic bacteria [33], and this would be one of the advantages of consuming chitin or its derivative chitosan together with probiotics to improve gut health. The probiotic bacteria already existing in the gut would benefit. There is currently more consciousness in functional food [34] and chitin from insects is likely to be a part of this. The use of insect chitin in the manufacture of an edible film should be encouraged owing to the importance of chitin and its role in modifying gut health.

Apart from nutrient depletion, there would be more metabolites produced by the growing probiotic bacteria, which would see a faster elimination of *Salmonella typhi*. Quite likely, this is why at 12 h the suppression of *Salmonella typhi* was high and with an increase in probiotic bacteria at 0%, 1%, and 5% chitosan concentrations. The presence of *Salmonella typhi* and high chitosan concentration beyond 5% did not favour the growth of *Lactobacillus acidophilus*. Despite the initial chitosan concentration, pathogenic bacteria were outgrown at the end, a likely result of the combined effects of the probiotic bacteria and low pH. Studies in fish fed with chitin showed increased survival, although there was no evidence of increased cellular immunity. The researchers pointed out that increased survival may have been the result of the suppression of pathogenic bacteria by chitin [35].The growth of probiotic bacteria may also have aided the suppression of pathogenic bacteria through the production of bacteriocin, exopolysaccharides, and biogenic amines [36]. These points to the importance of using chitin and chitosan as a prebiotic, and thus our findings encourage chitin or chitosan consumption to suppress pathogenic bacteria in the gut.

### 4.3. Prebiotics and Gut Health

Good prebiotic candidates would selectively support the growth of specific beneficial bacteria leading to a positive modulation of gut microbiota and according to Slomka et al. [37] in a study that was investigating the use of prebiotics in oral health, prebiotics greatly increased the proportion of beneficial but lowered that of pathogenic species. Pathogenic bacteria in the gut are a major cause of diarrhoea and the use of probiotic bacteria and chitosan that can suppress pathogenic bacteria by nourishing the probiotic species, which produce metabolites to kill off pathogenic species, has to be seen as an advance. Chitosan can also act directly as an antimicrobial in the human gut, suppressing the growth of pathogenic bacteria [33]. Many children in developing countries suffer greatly from childhood diarrhoea [38]. The addition of beneficial fibre such as chitin in the intervention that is aimed at helping children from the poor setup is likely to be more fruitful due to the additional benefits of chitin to gut health. Several antibacterial mechanisms of chitosan that have been proposed include: ionic surface interaction resulting in wall cell leakage; inhibition of the mRNA and protein synthesis via the penetration of chitosan into the nuclei of the microorganisms; formation of an external barrier, chelating metals and provoking the suppression of essential nutrients to microbial growth. It is likely that all events occur simultaneously but at different intensities [39].

The pH in chitosan containing *Salmonella typhi* growth media barely changed and was almost neutral at all chitosan concentrations (*p* > 0.05), but in *L. fermentum*, *B. adolescentis,* and *L. acidophilus* the pH decreased as the bacteria grew (Figure 3). In *L. acidophilus*, a species that seemed to thrive well at low pH, the highest drop in pH was noticed when the bacteria were growing in chitosan. This species was able to grow at a pH as low as pH 3.6, occurring in 20% chitosan-supplemented media at 24 h. All other probiotic bacteria growth media pH drops were seen after 12 h of growth, even in unmodified media. At 20%, chitosan concentration the expected pH drop was delayed up to 24 h (Figure 3). This might have been due to the slow growth of probiotic bacteria at high chitosan concentrations. A lower pH during growth is likely to be part of a survival strategy for probiotic bacteria and the lack to cope with the low pH in *Salmonella typhi* could be the reason why probiotic bacteria were able to suppress pathogenic bacteria. Fermented milk products have been consumed with perceived health benefits due to the presence of live bacteria responsible for milk fermentation, and recent years have seen an increased interest in fermented milk products due to their health benefit [40]. Combining fermented milk products with a prebiotic is likely to increase the benefits of its consumption.

Studies have shown that the antimicrobial activity of chitosan is pH-dependent since chitosan is soluble in an acidic environment. Yang et al., 2005 observed that the antibacterial activity of the N-alkylated chitosan derivatives against *E. coli* increased as the pH increased. These results verify that positive charge on the amino groups is not the sole factor resulting in antimicrobial activities because little is known about the antimicrobial activity of chitosan under alkaline conditions [41,42]

### 4.4. Altered Growth and Limitations

Nutrient limitations and a low pH have been indicated as the main inhibitors of the growth of pathogenic bacteria in the gut [2]. At the same time, they may have stimulated the growth of probiotic bacteria adapted to a low pH as such an adaptation is a well-documented survival strategy of many bacterial species [43]. Consumption of prebiotics would increase the colonization by probiotic bacteria, which in turn would suppress pathogenic bacteria and enhance their important role [44,45]. The gut microbiota would be highly regulated and therefore alleviate infections caused by microbial imbalances in the gut [46]. Intestinal microbiota depends on non-digestible fibre, [27] and cricket chitin is a possible prebiotic candidate. Fermentation of chitin leads to lower colonic pH due to the production of acetate, propionate, and butyrate by probiotic bacteria and these weak acids then influence the microbial composition, by suppressing pathogens, favouring the growth of probiotic bacteria [33,47,48]. The difference in the restriction distance was statistically significant at *p* < 0.05 for *L. fermentum* at 10% and 20% chitosan concentrations, for *B. adolescentis* at 10% and 20% chitosan concentrations, and for *L. acidophilus* at 5%, 10%, and 20% chitosan concentrations. In the media that did not contain chitosan, the difference was not significant *p* > 0.05. The presence of chitosan leads the bacteria to use the products of fermentation for their growth. During fermentation, weak acids and other metabolites restrict the growth of *Salmonella typhi.* There is a need to profile the metabolites in the media to ascertain the exact cause of pathogenic bacterial growth restriction, and the possibility of their further exploitation in the pharmaceutical industry.

Chitosan fermentation metabolites are known to inhibit bacterial growth [36,40,48,49], while chitosan itself exhibits antimicrobial activities that are useful in inhibiting gram-positive bacteria [50]. In a recent study, chitosan was shown to inhibit all of the bacterial strains tested [51]. Chitin hydrogels and other products have been developed to help in wound dressing and antimicrobial properties and to enhance healing [52]. In connection with the acceptance of insects as food and insect farming, suggested as early as 1975 [53], there is a need to fully utilise the chitin from these farmed insects in addition to the insects’ high protein content.

Apart from the antimicrobial potential of chitosan, it has other applications in the food industry that have been widely discussed by a review on the application of chitosan for improvement of quality and shelf life of food [54]. The antimicrobial activity of chitosan against a wide range of food-borne filamentous fungi, yeast, and bacteria has made it a potential food preservative. Chitosan also possesses film-forming and barrier properties, thus making it a potential raw material for edible films or coatings. Inherent antibacterial/antifungal properties and the film-forming ability of chitosan make it ideal for use as a biodegradable antimicrobial packaging material that can be used to improve the storability of perishable food [54]. Numerous researches have demonstrated that chitosan can be used as an effective preservative or coating material for the improvement of quality and shelf life of various foods [55,56,57,58,59]. Recently, the research on chitosan has increased drastically because of its considerable potential owing to its antimicrobial activity, biodegradability, non-toxicity, biocompatibility, and versatile chemical and physical properties as well as abundance. Chitosan has a significant role in the health and food application area, given the growing concern regarding the negative environmental impact of materials, deemed toxic, currently in use such as vinyl chlorides in plastic [59]. Chitosan-based polymeric materials can be formed into films, fibres, gels, sponges, nanoparticles, or even beads [60].

The most important food applications of chitosan include the encapsulating material for probiotic stability in the production of functional food products and the formation of biodegradable films and enzyme binding [61]. Probiotics’ mainly lactic acid bacteria (LAB) are widely used in the production of fermented dairy foods. They include yogurt and cheese and are the richest sources of probiotic foods [60,61]. For the probiotic microencapsulation technique used in connection with fruit juices, cereal-based products, chocolates, and cookies, chitosan is an ideal candidate as a coating material, since it does not affect the sensory properties of the encapsulated food [58,59,60,62].

## 5. Conclusions

Cricket chitin has a close similarity to commercial shrimp chitin. This study has demonstrated the ability of probiotic bacteria to break down chitosan, to lower the pH of growth media and thereby inhibit bacterial growth. Cricket-derived chitosan may be a functional prebiotic due to its ability to stimulate the growth of specific beneficial bacteria. Cricket-derived chitosan can help solve gut health in children directly by acting as an antimicrobial substance, or as a prebiotic to nourish probiotic bacteria.

## Figures and Tables

**Table 1:** Inhibition of pathogenic bacteria growth by probiotic bacteria supernatant derived from the growth of probiotic in chitosan-supplemented media.

	Bacterial Species		
Chitosan Concentration	*L. fermentum*	*B. adolescentis*	*L. acidophilus*
0%	10 ± 0.3 ^a^	10 ± 0.2 ^a^	10 ± 0.3 ^a^
1%	11 ± 0.3 ^a^	10 ± 0.3 ^a^	11 ± 0.2 ^a^
5%	11 ± 0.3 ^a^	10 ± 0.1 ^a^	12 ± 0.2 ^b^
10%	16 ± 0.3 ^b^	12 ± 0.2 ^b^	12 ± 0.3 ^b^
20%	13 ± 0.3 ^c^	14 ± 0.3 ^c^	10 ± 0.1 ^a^

*n* = 3. Values displayed as means ± standard deviation Values within the same column under the same chitosan concentration with different superscripts are significantly different *p* < 0.05 Abbreviation: *L. Fermentum*: *Lactobacillus fermentum*, *B. adolescentis*: *Bifidobacterium adolescentis*, *L. acidophilus*: *Lactobacillus acidophilus.* Bacterial growth in 0%, 1%, 5%, 10% and 20% chitosan-supplemented media was monitored at 24 h. Bacterial inhibition zones were measured in millimeters’ (mm).

## Data Availability

All data and materials are available on request.

## References

[1] Meyer-Rochow V.B., Sampat G., Jung C. (2019). Farming of insects for food and feed in South Korea: Tradition and innovation. Berl. und Münchener Tierärztliche Wochenschr..

[2] Di Gioia D., Biavati B. (2018). Probiotics and Prebiotics in Animal Health and Food Safety: Conclusive Remarks and Future Perspectives, in Probiotics and Prebiotics in Animal Health and Food Safety.

[3] Seabrooks L., Hu L. (2017). Insects: An underrepresented resource for the discovery of biologically active natural products. Acta Pharm. Sin. B.

[4] Paoletti M.G., Norberto L., Damini R., Musumeci S. (2007). Human gastric juice contains chitinase that can degrade chitin. Ann. Nutr. Metab..

[5] Tian B., Liu Y. (2020). Chitosan-based biomaterials: From discovery to food application. Polym. Adv. Technol..

[6] Aranaz I., Acosta N., Civera C., Elorza B., Mingo J., Castro C., Gandía M.D.L.L., Caballero A.H. (2018). Cosmetics and cosmeceutical applications of chitin, chitosan and their derivatives. Polymers.

[7] Wang W., Xue C., Mao X. (2020). Chitosan: Structural modification, biological activity and application. Int. J. Biol. Macromol..

[8] Ibram A., Ionescu A.-M., Cadar E. (2021). Comparison of extraction methods of chitin and chitosan from different sources. Eur. J. Med. Nat. Sci..

[9] Mlcek J., Borkovcová M., Bednářová M. (2014). Biologically active substances of edible insects and their use in agriculture, veterinary and human medicine. J. Cent. Eur. Agric..

[10] Gibson G.R., Hutkins R., Sanders M.E., Prescott S.L., Reimer R.A., Salminen S.J., Scott K., Stanton C., Swanson K.S., Cani P.D. (2017). Expert consensus document: The International Scientific Association for Probiotics and Prebiotics (ISAPP) consensus statement on the definition and scope of prebiotics. Nat. Rev. Gastroenterol. Hepatol..

[11] Vogt S.L., Finlay B.B. (2017). Gut microbiota-mediated protection against diarrheal infections. J. Travel Med..

[12] Kazemi A., Noorbala A.A., Azam K., Eskandari M.H., Djafarian K. (2019). Effect of probiotic and prebiotic vs placebo on psychological outcomes in patients with major depressive disorder: A randomized clinical trial. Clin. Nutr..

[13] Stull V.J., Finer E., Bergmans R.S., Febvre H.P., Longhurst C., Manter D.K., Patz J.A., Weir T.L. (2018). Impact of edible cricket consumption on gut microbiota in healthy adults, a double-blind, randomized crossover trial. Sci. Rep..

[14] Radke M., Picaud J.-C., Loui A., Cambonie G., Faas D., Lafeber H.N., De Groot N., Pecquet S.S., Steenhout P.G., Hascoet J.-M. (2017). Starter formula enriched in prebiotics and probiotics ensures normal growth of infants and promotes gut health: A randomized clinical trial. Pediatr. Res..

[15] Magara H., Tanga C., Ayieko M., Hugel S., Mohamed S.A., Khamis F., Salifu D., Niassy S., Subramanian S., Fiaboe K.K.M. (2019). Performance of newly described native edible cricket *Scapsipedus icipe* (Orthoptera: Gryllidae) on various diets of relevance for farming. J. Econ. Entomol..

[16] Sørensen J., Loeschcke V. (2001). Larval crowding in Drosophila melanogaster induces Hsp70 expression, and leads to increased adult longevity and adult thermal stress resistance. J. Insect Physiol..

[17] Otieno M.H.J., Ayieko M.A., Niassy S., Salifu D., Abdelmutalab A.G.A., Fathiya K.M., Subramanian S., Fiaboe K.K.M., Roos N., Ekesi S. (2019). Integrating temperature-dependent lifetable data into insect life cycle model for predicting the potential distribution of Scapsipedus icipe Hugel & Tanga. PLoS ONE.

[18] Ryan M.P., Rea M.C., Hill C., Ross R. (1996). An application in cheddar cheese manufacture for a strain of Lactococcus lactis producing a novel broad-spectrum bacteriocin, lacticin 3147. Appl. Environ. Microbiol..

[19] Goy R.C., Morais S.T., Assis O.B. (2016). Evaluation of the antimicrobial activity of chitosan and its quaternized derivative on *E. coli* and *S. aureus* growth. Rev. Bras. Farm..

[20] Motulsky H. (1998). The GraphPad Guide to Comparing Dose-Response or Kinetic Curves.

[21] Atay H.Y. (2019). Antibacterial activity of chitosan-based systems. Funct. Chitosan.

[22] Martău G.A., Mihai M., Vodnar D.C. (2019). The use of chitosan, alginate, and pectin in the biomedical and food sector—biocompatibility, bioadhesiveness, and biodegradability. Polymers.

[23] Kumar M., Vivekanand V., Pareek N. (2020). Insect Chitin and Chitosan: Structure, Properties, Production, and Implementation Prospective, in Natural Materials and Products from Insects: Chemistry and Applications.

[24] Zainol Abidin N.A., Kormin F., Zainol Abidin N.A., Mohamed Anuar N.A.F., Abu Bakar M.F. (2020). The potential of insects as alternative sources of chitin: An overview on the chemical method of extraction from various sources. Int. J. Mol. Sci..

[25] Lee H., Zandkarimi F., Zhang Y., Meena J.K., Kim J., Zhuang L., Tyagi S., Ma L., Westbrook T.F., Steinberg G.R. (2020). Energy-stress-mediated AMPK activation inhibits ferroptosis. Nat. Cell Biol..

[26] Anal A.K., Singh H. (2007). Recent advances in microencapsulation of probiotics for industrial applications and targeted delivery. Trends Food Sci. Technol..

[27] Sawicki C.M., Livingston K.A., Obin M., Roberts S.B., Chung M., McKeown N.M. (2017). Dietary fiber and the human gut microbiota: Application of evidence mapping methodology. Nutrients.

[28] Jarett J.K., Carlson A., Serao M.R., Strickland J., Serfilippi L., Ganz H.H. (2019). Diets with and without edible cricket support a similar level of diversity in the gut microbiome of dogs. PeerJ.

[29] Marzorati M., Maquet V., Possemiers S. (2017). Fate of chitin-glucan in the human gastrointestinal tract as studied in a dynamic gut simulator (SHIME^®^). J. Funct. Foods.

[30] Buruiana C.-T., Gómez B., Vizireanu C., Garrote G. (2017). Manufacture and evaluation of xylo-oligosaccharides from corn stover as emerging prebiotic candidates for human health. LWT.

[31] Álvarez-Ordóñez A., Begley M., Prieto M., Messens W., López M., Bernardo A., Hill C. (2011). Salmonella spp. survival strategies within the host gastrointestinal tract. Microbiology.

[32] Bäumler A.J., Sperandio V. (2016). Interactions between the microbiota and pathogenic bacteria in the gut. Nature.

[33] Satokari R. (2019). Modulation of gut microbiota for health by current and next-generation probiotics. Nutrients.

[34] Díaz L.D., Fernández-Ruiz V., Cámara M. (2020). An international regulatory review of food health-related claims in functional food products labeling. J. Funct. Foods.

[35] Dong L., Ariëns R.M.C., Tomassen M.M., Wichers H.J., Govers C. (2020). In vitro studies toward the use of chitin as nutraceutical: Impact on the intestinal epithelium, macrophages, and microbiota. Mol. Nutr. Food Res..

[36] Prodeus A., Niborski V., Schrezenmeir J., Gorelov A., Shcherbina A., Rumyantsev A. (2016). Fermented milk consumption and common infections in children attending day-care centers: A randomized trial. J. Pediatr. Gastroenterol. Nutr..

[37] Slomka V., Herrero E.R., Boon N., Bernaerts K., Trivedi H.M., Daep C. (2018). Oral prebiotics and the influence of environmental conditions in vitro. J. Peridontol..

[38] Platts-Mills J.A., Liu J., Rogawski E.T., Kabir F., Lertsethtakarn P., Siguas M., Khan S.S., Praharaj I., Murei A., Nshama R. (2018). Use of quantitative molecular diagnostic methods to assess the aetiology, burden, and clinical characteristics of diarrhoea in children in low-resource settings: A reanalysis of the MAL-ED cohort study. Lancet Glob. Health.

[39] Goy R.C., de Britto D., Assis O.B.G. (2009). A review of the antimicrobial activity of chitosan. Polímeros.

[40] Vasiljevic T., Shah N.P. (2007). Fermented milk: Health benefits beyond probiotic effect. Handbook of Food Products Manufacturing.

[41] Su Z., Han Q., Zhang F., Meng X., Liu B. (2020). Preparation, characterization and antibacterial properties of 6-deoxy-6-arginine modified chitosan. Carbohydr. Polym..

[42] Yang T.-C., Chou C.-C., Li C.-F. (2005). Antibacterial activity of N-alkylated disaccharide chitosan derivatives. Int. J. Food Microbiol..

[43] Singh R., Mishra N.K., Kumar V., Vinayak V., Joshi K.B. (2018). Transition metal ion-mediated tyrosine-based short-peptide amphiphile nanostructures inhibit bacterial growth. ChemBioChem.

[44] Hojsak I. (2019). Probiotics in functional gastrointestinal disorders. Adv. Exp. Med. Biol..

[45] Vandenplas Y., Savino F. (2019). Probiotics and prebiotics in pediatrics: What is new?. Nutrients.

[46] Gao J., Xu K., Liu H., Liu G., Bai M., Peng C., Li T., Yin Y. (2018). Impact of the gut microbiota on intestinal immunity mediated by tryptophan metabolism. Front. Cell. Infect. Microbiol..

[47] Galdeano C.M., Cazorla S.I., Dumit J.M.L., Vélez E., Perdigón G. (2019). Beneficial effects of probiotic consumption on the immune system. Ann. Nutr. Metab..

[48] Jeong C.-W., Choi H.-J., Yoo G.-Y., Lee S.-H., Kim Y.-C., Okorie O.E., Lee J.-H., Jun K.-D., Choi S.-M., Kim K.-W. (2006). Effects of dietary probiotics supplementation on juvenile Olive Flounder *Paralichthys olivaceus*. Korean J. Fish. Aquat. Sci..

[49] Moreno-Vásquez M.J., Valenzuela-Buitimea E.L., Plascencia-Jatomea M., Encinas J.C., Rodríguez-Félix F., Sánchez-Valdes S., Rosas-Burgos E., Ocaño-Higuera V.M., Graciano-Verdugo A.Z. (2017). Functionalization of chitosan by a free radical reaction: Characterization, antioxidant and antibacterial potential. Carbohydr. Polym..

[50] Cremar L., Gutierrez J., Martinez J., Materon L., Gilkerson R., Xu F., Lozano K. (2018). Development of antimicrobial chitosan based nanofiber dressings for wound healing applications. Nanomed. J..

[51] Abdelmalek B.E., Sila A., Haddar A., Bougatef A., Ayadi M.A. (2017). β-Chitin and chitosan from squid gladius: Biological activities of chitosan and its application as clarifying agent for apple juice. Int. J. Biol. Macromol..

[52] Shao W., Wu J., Ye S., Wang S., Jiang L., Wei S., Jimin W., Shan Y., Shuxia W., Lei J. (2017). A facile and green method to prepare chitin based composites with antibacterial activity. J. Bionanosci..

[53] Meyer-Rochow V.B. (1975). Can insects help to ease the probem of world food shrtage?. Search.

[54] Chien P.-J., Sheu F., Lin H.-R. (2007). Coating citrus (Murcott tangor) fruit with low molecular weight chitosan increases postharvest quality and shelf life. Food Chem..

[55] Butnaru E., Stoleru E., Brebu M.A., Darie-Nita R.N., Bargan A., Vasile C. (2019). Chitosan-Based bionanocomposite films prepared by emulsion technique for food preservation. Materials.

[56] Noorbakhsh-Soltani S., Zerafat M., Sabbaghi S. (2018). A comparative study of gelatin and starch-based nano-composite films modified by nano-cellulose and chitosan for food packaging applications. Carbohydr. Polym..

[57] Kumar S., Mukherjee A., Dutta J. (2020). Chitosan based nanocomposite films and coatings: Emerging antimicrobial food packaging alternatives. Trends Food Sci. Technol..

[58] Freepons D. (2020). Enhancing food production with chitosan seed-coating technology. Applications of Chitin and Chitosan.

[59] da Costa J.C.M., Miki K.S.L., da Silva Ramos A., Teixeira-Costa B.E. (2020). Development of biodegradable films based on purple yam starch/chitosan for food application. Heliyon.

[60] Piotrowska B. (2005). Toxic Components of Food Packaging Materials.

[61] Kritchenkov A.S., Egorov A., Yagafarov N.Z., Volkova O.V., Zabodalova L.A., Suchkova E.P., Kurliuk A.V., Khrustalev V. (2020). Efficient reinforcement of chitosan-based coatings for Ricotta cheese with non-toxic, active, and smart nanoparticles. Prog. Org. Coat..

[62] Călinoiu L.F., Vodnar D.C. (2020). Thermal processing for the release of phenolic compounds from wheat and oat bran. Biomolecules.

